# Association of Travel Time and Residential Location With the Use of Antenatal Care and Institutional Delivery Services in Afghanistan

**DOI:** 10.1155/ogi/1366466

**Published:** 2026-01-28

**Authors:** Massoma Jafari, Essa Tawfiq, Muhammad Haroon Stanikzai, Sheena Currie, Fatima Arifi, Faiza Rab, Hawa Kazemi, Abdul Wahed Wasiq, Sabera Turkmani

**Affiliations:** ^1^ Department of Health Profession Education Research, University of Toronto, Toronto, Ontario, Canada, utoronto.ca; ^2^ The Kirby Institute, UNSW Sydney, Sydney, New South Wales, Australia, unsw.edu.au; ^3^ Department of Public Health, Faculty of Medicine, Kandahar University, Kandahar, Afghanistan, kdru.edu.af; ^4^ Afghan Midwives Association, Kabul, Afghanistan; ^5^ Independent Researcher, Toronto, Ontario, Canada; ^6^ Department of Epidemiology and Biostatistics, Western University, London, Ontario, Canada, uwo.ca; ^7^ Department of General Medicine, Kateb University, Kabul, Afghanistan; ^8^ Department of Internal Medicine, Faculty of Medicine, Kandahar University, Kandahar, Afghanistan, kdru.edu.af; ^9^ Faculty of Health, University of Technology Sydney, Sydney, New South Wales, Australia, uts.edu.au

**Keywords:** Afghanistan, ANC, health-facility delivery, rural residence, travel time, urban residence

## Abstract

**Background:**

Equitable access to maternal healthcare hinges on overcoming logistical and socio‐economic challenges in many low‐ and middle‐income countries (LMICs).

**Objectives:**

This study examines the association of travel time to health facilities and residential areas with the use of antenatal care (ANC) and institutional delivery services in Afghanistan.

**Methods:**

We used data from the Afghanistan Health Survey 2018, focusing on 1051 ever‐married women aged 15–49 who had recently given birth and had ≥ 1 ANC session. The study measured the association of travel time and residential location, along with sociodemographic characteristics, on two primary outcomes: institutional deliveries and ANC service frequency. A generalized linear model facilitated the multivariable regression analyses.

**Results:**

The study found that travel time to health facilities and residential locations significantly influenced ANC utilization and institutional deliveries. Women with travel time of 0.5–2 and < 0.5 h to health facilities received 78% and 65% more ANC visits, respectively, than women with travel time of > 2 h to health facilities. Women who lived in rural areas received 50% fewer ANC visits compared to women who lived in urban areas. Women with travel time of 0.5–2 h were more likely to have institutional deliveries (odds ratio [OR] = 2.56, 95% confidence interval [CI]; 1.43–4.59) than those with travel time of > 2 h to health facilities. The likelihood of institutional deliveries was lower among rural resident women (OR = 0.62, 95% CI; 0.40–0.97) than their urban counterparts. Other predictors of ANC visits were women’s education level and women’s knowledge of complicated pregnancy, and other predictors of institutional deliveries were women’s education level, decisions made for women about birthplace choice, and women’s access to media.

**Conclusion:**

We have shown for the first time that access to health facilities and geographic disparities significantly influence maternal health service utilization in Afghanistan.

## 1. Introduction

Achieving the Sustainable Development Goals (SDGs), particularly reducing the maternal mortality ratio (MMR) to less than 70 per 100,000 live births and ending preventable deaths for newborns and children under five years of age, remains a challenge in Afghanistan [1]. The provision of skilled birth attendance (SBA) and comprehensive antenatal care (ANC) are vital for safe childbirth and improving maternal and neonatal health outcomes [1, 2]. Low quality and less frequent ANC are associated with a decreased utilization of facility‐based deliveries essential for reducing maternal and neonatal mortality [3, 4]; however, access to these essential services remains inconsistent [1]. Evidence highlights disparities in the use of maternal healthcare, influenced by factors such as wealth, education, residence, religion, and decision‐making power, resulting in inequalities within and between nations [1, 5].

Compared to wealthier, urban women, those in rural areas face access‐related challenges, exacerbated by logistical challenges such as distances to medical centers and time required to reach them [6–8]. A study from Ghana reported that each additional kilometer in the distance markedly decreased the rate of women delivering in health facilities by 6.7% [6].

Adding to these logistical issues, the social standing of women, including their education levels, understanding of maternal health issues, knowledge about healthcare options, and access to media for health information, also play a pivotal role in the uneven utilization of maternal healthcare services [9]. Education informs health choices [10], and cultural norms influence service use [11, 12]. Women’s autonomy also affects their healthcare utilization [13].

Afghanistan stands at the forefront of this challenge, historically bearing a disproportionately high burden of maternal and neonatal deaths [14]. While significant improvements were made between 2000 and 2017, with a 56% reduction in MMR from 1450 to 638 per 100,000 live births [15, 16], the journey toward further progress is complicated by political upheaval and escalating humanitarian crises.

Timely quality ANC provides an essential window to “avert, identify, and manage” potential pregnancy complications by the provision of essential maternal health services [17]. In Afghanistan, only 28% of women receive the previously recommended 4+ ANC visits [18–20]. Moreover, the institutional birth rate is 58.8% [21, 22].

Afghanistan has made commendable efforts toward increasing access to health services, including institutional deliveries and ANC services, through implementing the Basic Package of Health Services (BPHS) and the Essential Package of Hospital Services (EPHS) with the expansion of health facilities from 498 in 2002 to 3135 in 2019 [16]. Expanding community healthcare, strengthened by community midwifery education initiatives and establishing family health houses, has been crucial in improving access to maternal health services [23, 24].

In the intricate context of Afghanistan, the “three delays” model—identifying the need for care, reaching healthcare facilities, and receiving adequate care upon arrival—remains a critical analytical framework for understanding the persistent challenges in reducing maternal and newborn mortality [25]. The harsh climate, mountainous terrain, and inadequate transportation exacerbate a weak referral system. Urbanization, poor health infrastructure, economic instability, insecurity, and the effects of drought further intensify these issues [26]. Large distances to, and quality of care at, health facilities are critical, as they directly influence maternal and newborn mortality, often causing women to bypass closer, lower‐quality facilities [27].

In Afghanistan, the healthcare system has long been facing several challenges [28]. These challenges are compounded by the collapse of the Afghan Government and the health system’s heavy reliance on humanitarian aid, which comprises 75% of the nation’s total public expenditure, including health‐related costs [29]. Following the political transition and subsequent pause in financial support from the World Bank and other donors, reduced health sector funding threatens to reverse the significant gains achieved [26]. The sustainability of healthcare is critically dependent on international support, making it vulnerable to geopolitical shifts and policy changes. Over a consistent four‐month period spanning three years, data from the Ministry of Public Health (MoPH) indicated a 21% decrease in utilization of ANC services in 2021, relative to 2020 [16]. Additionally, there was a notable 29% decline in institutional delivery during September 2021 compared to the same month in 2020 [16]. This decrease occurred simultaneously with heightened conflict and corresponding disruptions in health services (due to the COVID‐19 pandemic) [16].

Our research aims to evaluate the association of travel time and residential locations with the use of ANC and institutional delivery services. By identifying patterns and predictors of healthcare access from the 2018 national survey, this study not only reflects on past conditions but also highlights the urgency of informed interventions in the current context, making it a pivotal contribution to the discourse on maternal healthcare in Afghanistan.

## 2. Methods

### 2.1. Data Source

We used information gathered for the 2018 Afghanistan Health Survey (AHS) for this cross‐sectional research [21]. The survey design for the AHS2018 included 23,460 randomly selected households, divided over 1020 clusters in 34 provinces, and data were collected from 19,684 randomly selected households, divided over 912 clusters in 34 provinces. A stratified two‐stage cluster sampling approach was undertaken for the AHS2018. In each province, stratification was done by urban and rural areas. In each stratum, at the first stage, a household listing activity was carried out in all 1020 randomly selected clusters by visiting each of the selected clusters to prepare a list of occupied residential households. In the second stage, within the stratum, the list of occupied residential households was used as the sampling frame, and for each cluster, 23 households were randomly selected via a systematic sampling approach. The list of selected households was provided to the surveyors to conduct the survey. To minimize the occurrence of selection bias, no replacements were allowed in the selected households. The survey design of the AHS2018 has been described in detail elsewhere [21]. Female data collectors with experience in this area questioned women in the homes that were chosen to participate. They used a standardized questionnaire to gather information about how often women and children utilized health services.

From a total of 9469 ever‐married women, aged under 50 years, who gave birth in the past 2 years and attended at least one ANC session, 1051 women met our inclusion criteria and enrolled in this study (Figure 1). As of the AHS 2018, “ever‐married” includes those married at the time of the survey, divorced, widowed, or single women caring for a child under five [21]. We used and analyzed data with complete information, which means a listwise deletion approach was used and data with missing values were excluded from analysis (Figure 1). The reason that we used complete cases and did not impute missing values on travel time relates to the existence of a large amount of missing information from 4992 women (52.7% of 9469 ever‐married women interviewed). Since we excluded 4992 women with missing values on travel time, we examined the distribution of sociodemographic characteristics and use of ANC services by type of health workers between the 4992 women and those 1051 women we included in the study, stratified by institutional versus non‐institutional deliveries (see supporting information). As can be seen in the supporting information, the distribution of sociodemographic characteristics and use of ANC services by type of health workers between women with complete information on travel time and women with missing values on travel time does not show considerable differences. For example, 22.7% of 1051 versus 26.6% of 4992 women lived in urban areas, 37.4% of 1051 versus 30.7% of 4992 women received ANC services from doctors, and 61.9% of 1051 versus 67.9% of 4992 women received ANC services from midwives, for women with complete information on travel time versus women with missing values on travel time, respectively. Thus, the use of data from 1051 women in this study may not have considerably compromised the external validity of the study.

**Figure FIGURE 1:**
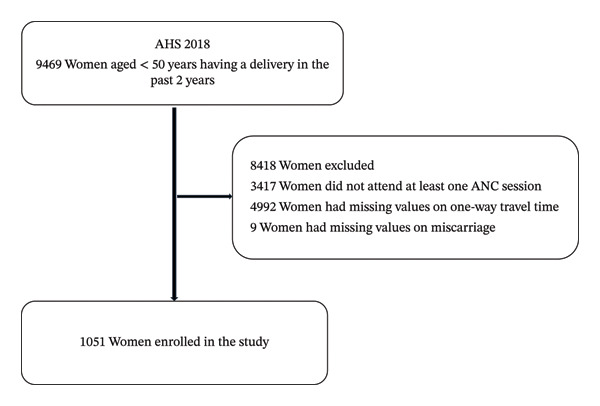
Final sample size and schematic presentation of the sample selection.

### 2.2. Study Variables

#### 2.2.1. Outcome variables

We examined two outcomes. The first outcome was the number of ANC visits (continuous variable), defined as the number of times an ever‐married pregnant woman attended ANC services offered by a health worker (community health worker, nurse/midwife, or doctor), and the second outcome was institutional delivery (binary variable), defined as a birth within the 2 years that an ever‐married woman had in a health facility.

#### 2.2.2. Predictors

Guided by the literature on maternal health and access to health facilities, we selected the following predictors (covariates) and included them in the statistical model [9, 10, 12, 18, 19]. The main predictors of interest were the one‐way travel time to a health facility (< 0.5, 0.5–2, and > 2 h) and the residential area (urban vs. rural) that an ever‐married woman reported. Other predictors of interest considered in the study’s statistical analyses were woman’s age (15–29, 30–34, and 35–49 years), woman’s education level (no formal education, primary/intermediate, and secondary/higher education), woman’s knowledge of pregnancy danger signs (had knowledge vs. did not have knowledge), woman’s decision‐making as to where to give birth (the decision made by herself, by her husband, by her in‐laws, and by others/friends), whether the women had a miscarriage in the past (yes vs. no), woman’s access to the media (whether she had access to the internet, radio, or TV daily or weekly), and the type of health worker who provided ANC services (community health worker, midwife/nurse, or doctor). We did not include a variable on socioeconomic status in the analyses because it was highly correlated with woman’s education, woman’s decision‐making, and woman’s knowledge of danger signs related to pregnancy, and the inclusion of socioeconomic status could have biased the multivariable results.

### 2.3. Statistical Analysis

Descriptive statistics were used to describe the outcome and predictor variables. A simple linear regression model was used to examine the number of ANC visits (a continuous variable), and the binary logistic regression model was used to examine institutional deliveries (a binary variable). Mean differences in the number of ANC visits were used to assess the effect of the first outcome. Unadjusted and adjusted odds ratios (ORs) were used to compare institutional versus non‐institutional deliveries. Given that data were gathered at the level of each household, we included a random cluster effect in our model estimates. This was done to account for the clustering effects of the data at the household level and to adjust the standard errors for the ORs, coefficients, and their related 95% confidence intervals (CIs). The data analysis was conducted using STATA Version 17.

### 2.4. Ethical Approval

The study was reviewed by the Research and Ethics Committee, Department of Public Health, Faculty of Medicine, Kandahar University, Afghanistan. The committee waived ethical approval for this study since it solely utilized and analyzed existing secondary data from the AHS2018.

## 3. Results

Most (62.2%) of women with a 0.5–2 h journey chose institutional births. In rural areas, 85.6% opted for non‐institutional, while 73.9% chose institutional deliveries. Overall, most ANC services were delivered by midwives (61.9%) and doctors (37.4%), a distribution that was similar for institutional and non‐institutional deliveries (Table 1).

**Table TABLE 1:** Access attributes to travel time to health facilities and baseline characteristics of women.

Characteristics	Non‐institutional deliveries	Institutional deliveries	Total
	*n* = 298 (%)	*n* = 753 (%)	*n* = 1051 (%)
^∗∗∗^One‐way travel time to a health facility			
> 2 h	10.4	6.3	7.4
0.5–2 h	44.0	62.2	57.0
< 0.5 h	45.6	31.6	35.6
Residential area			
Urban	14.4	26.1	22.8
Rural	85.6	73.9	77.2
Health worker who provided ANC services			
Community Health Worker	0.7	0.1	0.3
Nurse	0.0	0.5	0.4
Doctor	33.9	38.8	37.4
Midwife	65.4	60.6	61.9
Age of woman			
15–29 years	58.1	55.3	56.1
30–34 years	24.2	22.2	22.8
35–49 years	17.8	22.5	21.2
^∗^Woman’s education level			
No formal education	80.2	76.4	77.5
Primary	15.8	14.8	15.1
Secondary	4.0	8.8	7.4
Woman knows danger sign			
No	35.9	32.2	33.3
Yes	64.1	67.8	66.7
^∗∗∗^Decision made for woman on where to give birth			
Herself	59.4	25.7	35.3
Husband	24.2	48.7	41.8
In‐laws	8.1	21.8	17.9
Others	8.4	3.7	5.1
Woman had a miscarriage in the past			
No	64.4	66.7	66.1
Yes	35.6	33.3	33.9
^∗∗^Access to media			
No	53.0	41.8	45.0
Almost daily	25.8	36.0	33.1
Once a week	21.1	22.2	21.9

^∗^ *p*‐value < 0.05.

^∗∗^ *p*‐value < 0.01.

^∗∗∗^ *p*‐value < 0.001.

Regarding age, women aged 15–29 had more non‐institutional deliveries (58.1%) compared to 55.3% institutional, while women aged 35–49 showed a higher tendency for institutional births (22.5% vs. 17.8%). More than three‐quarters (76.4%) of women without formal education leaned toward non‐institutional deliveries (76.4%). Awareness of danger signs increases institutional births (67.8%).

In terms of decision‐making, the data revealed that among the women who had institutional deliveries, most of the time it was the husbands who were the decision‐makers (48.7%), followed by the women themselves (25.7%) and then the in‐laws (21.8%). This was in contrast to decision‐making when it came to non‐institutional deliveries, where the majority of the women themselves (59.4%) were the decision‐makers, followed by their husbands (24.2%). Only 33.3% of women who had miscarriages received institutional deliveries compared to 35.6% of non‐institutional deliveries. Moreover, the findings revealed that women with access to media had more institutional births, with 36.0% of women with daily media access choosing this option (Table 1).

Table 2 shows that on average (mean, median), women who lived in an urban area received more ANC visits than women who lived in a rural area. For example, the mean ANC visits for women in an urban area versus for women in a rural area were 4.2 versus 3.4 visits, respectively. The mean ANC visits in an urban area ranged from 5.0 to 4.1 to 4.5 visits for women with > 2 h, 0.5–2 h, and < 0.5 h, respectively. However, the mean ANC visits in a rural area ranged from 2.5 to 3.6 to 3.3 visits for women with > 2 h, 0.5–2 h, and < 0.5 h one‐way travel time to health facilities, respectively (Table 2).

**Table TABLE 2:** Average number of ANC sessions attended by women in urban and rural areas.

Residential area	*n*	Mean (SD)	Median
Urban area			
> 2 h	8	5.0 (1.2)	5.5
0.5–2 h	203	4.1 (2.5)	3.0
< 0.5 h	28	4.5 (2.8)	3.0
Subtotal	239	4.2 (2.5)	3.0
Rural area			
> 2 h	70	2.5 (1.6)	2.0
0.5–2 h	397	3.6 (2.3)	3.0
< 0.5 h	345	3.3 (2.1)	3.0
Subtotal	812	3.4 (2.2)	3.0
Total	1051	3.6 (2.3)	3.0

Unadjusted analysis showed a strong association between institutional deliveries and residential areas was observed with significantly lower odds of institutional deliveries for women living in a rural area (OR: 0.48 [0.33, 0.69]). For one‐way travel time of < 0.5 h, 0.5–2 h, and > 2 h, odds of institutional deliveries were lower for women living in a rural area as compared with those living in an urban area, but only significant for those with a travel time of 0.5–2 h (Table 3).

**Table TABLE 3:** Likelihood of having institutional deliveries by women in rural versus urban (reference) areas.

One‐way time to a health facility	*n*	Crude odds ratio	(95% CI)	*p*‐value
> 2 h	78	0.19	(0.02, 1.65)	0.13
0.5–2 h	600	0.65	(0.42, 1.00)	0.05
< 0.5 h	373	0.56	(0.21, 1.47)	0.24
Total	1051	0.48	(0.33, 0.69)	< 0.001

Table 4 shows the results from multivariate analysis on the number of ANC visits women received. Women with travel time of 0.5–2 h and < 0.5 h to health facilities received 78% (0.78 [95% CI; 0.34, 1.23]) and 65% (0.65 [95% CI; 0.19, 1.10]) more ANC visits than women with travel time of > 2 h to health facilities. Women who lived in a rural area received 50% fewer ANC visits (–0.50 [95% CI; −0.89, −0.12]) compared to women who lived in an urban area. Women who received ANC services from midwives had a 30% increase in visits (0.30 [95% CI; 0.02, 0.57]) compared to those who received ANC services from doctors. Women with primary/intermediate education and secondary/higher education received 72% and 93% more ANC visits, respectively, than those with no formal education (0.72 [95% CI; 0.27, 1.17]; 0.93 [95% CI; 0.29, 1.56]). The women who knew danger signs of pregnancy received 58% more ANC visits than those who did not know any danger symptoms (0.58 [95% CI; 0.30, 0.85]).

**Table TABLE 4:** Effects of accessibility and sociodemographic factors on use of ANC services (number of ANC visits).

Characteristics	Coefficients	(95% CI)	*p*‐value
One‐way travel time to a health facility			
> 2 h	Ref		
0.5–2 h	0.78	(0.34, 1.23)	< 0.001
< 0.5 h	0.65	(0.19, 1.10)	0.01
Residential area			
Urban	Ref		
Rural	−0.50	(−0.89, −0.12)	0.01
Health worker who provided ANC services			
Doctor	Ref		
Midwife	0.30	(0.02, 0.57)	0.04
^∗^Nurse/CHW	—	—	—
Age of woman			
15–29 years	Ref		
30–34 years	0.04	(−0.30, 0.38)	0.80
35–49 years	0.27	(−0.11, 0.64)	0.16
Woman’s education level			
No formal education	Ref		
Primary	0.72	(0.27, 1.17)	< 0.001
Secondary	0.93	(0.29, 1.56)	< 0.001
Woman knows danger symptoms			
No	Ref		
Yes	0.58	(0.30, 0.85)	< 0.001
Decision made for woman on where to give birth			
Herself	Ref		
Husband	0.23	(−0.10, 0.56)	0.17
In‐laws	−0.04	(−0.43, 0.34)	0.83
Others	−0.17	(−0.80, 0.46)	0.59
Woman had a miscarriage in the past			
No	Ref		
Yes	0.15	(−0.14, 0.44)	0.31
Access to media			
No access	Ref		
Almost daily	0.14	(−0.20, 0.48)	0.42
Once a week	0.11	(−0.25, 0.48)	0.54

^∗^There were 4 nurses and 3 CHWs; therefore, the coefficient for them was not obtained. Coefficients refer to the percentage of ANC visits in the categories of explanatory variables relative to the reference category.

Table 5 shows the results from multivariate analysis on the likelihood of institutional deliveries. The odds of institutional deliveries were higher for women with travel time 0.5–2 h (adjusted OR 2.56 [95% CI; 1.43, 4.59]) compared with women with travel time > 2 h to health facilities. The odds of institutional deliveries were lower for women who lived in a rural area (OR 0.62 [95% CI; 0.40, 0.97]) compared with women who lived in an urban area. Women aged 35–49 years had greater odds of institutional deliveries (OR 1.76 [95% CI; 1.14, 2.72]) compared with women aged 15–29 years. Women with secondary/higher education had higher odds of institutional deliveries (OR 2.14 [95% CI; 1.03, 4.44]) compared with women with no formal education. For women for whom their husbands and in‐laws made the decision of where to give birth, it was more likely to have institutional deliveries than the women who made the decision themselves (OR 6.12 [95% CI; 4.26, 8.77]; OR 8.20 [95% CI; 5.01, 13.42]), respectively. Women with daily access to media (the internet, radio, and TV) had higher odds of institutional deliveries as compared to women with no access to media (1.67 [95% CI; 1.15, 2.43]).

**Table TABLE 5:** Effects of accessibility and sociodemographic factors on institutional deliveries by women.

Characteristics	AOR	95% CI	*p*‐value
One‐way travel time to a health facility			
> 2 h	Ref		
0.5–2 h	2.56	(1.43, 4.59)	< 0.001
< 0.5 h	1.12	(0.62, 2.02)	0.70
Residential area			
Urban	Ref		
Rural	0.62	(0.40, 0.97)	0.04
Health worker who provided ANC services			
Doctor	Ref		
Midwife	0.97	(0.71, 1.35)	0.88
^∗^Nurse/CHW	—	—	—
Age of woman			
15–29 years	Ref		
30–34 years	1.16	(0.81, 1.68)	0.42
35–49 years	1.76	(1.14, 2.72)	0.01
Woman’s education level			
No formal education	Ref		
Primary	0.95	(0.63, 1.45)	0.83
Secondary	2.14	(1.03, 4.44)	0.04
Woman knows danger symptoms			
No	Ref		
Yes	1.26	(0.92, 1.73)	0.16
Decision made for woman on where to give birth			
Herself	Ref		
Husband	6.12	(4.26, 8.77)	< 0.001
In‐laws	8.20	(5.01,13.42)	0.001
Others	0.96	(0.52, 1.76)	0.89
Woman had a miscarriage in the past			
No	Ref		
Yes	1.03	(0.75, 1.42)	0.85
Access to media			
No access	Ref		
Almost daily	1.67	(1.15, 2.43)	0.01
Once a week	1.36	(0.91, 2.03)	0.14

^∗^There were 4 nurses and 3 CHWs; therefore, the odds ratio for them was not obtained. Adjusted odds ratios refer to the likelihood of institutional deliveries in categories of explanatory variables relative to the reference category.

## 4. Discussion

This study identifies the crucial influence of travel time to health facilities and women’s residential locations on utilization of ANC services and institutional deliveries in Afghanistan. Notably, women residing within 0.5–2 h of a health facility exhibit higher rates of institutional deliveries compared to those facing longer travel distances. This pattern aligns with observations in humanitarian settings, emphasizing the pivotal role of travel time and accessibility in health service utilization [6, 7]. A similar relationship between travel time to health facilities and increased institutional delivery and having a skilled birth attendant (SBA) has been reported in various settings [30, 31], such as Nigeria, reinforcing the importance of minimizing travel barriers for improved maternal health outcomes [32], which aligns with our findings.

In Afghanistan, maternal healthcare access reveals stark socioeconomic disparities. Only 28% of women achieve the recommended four or more ANC visits, with a mere 54% benefiting from SBAs [31]. The wealth gap exacerbates these discrepancies, showing a 25‐percentage‐point difference in ANC rate and a 61% contrast in the coverage of SBA between the poorest and wealthiest. Aligned with the AHS 2018 report, this study identifies travel time to healthcare facilities as a crucial determinant, revealing geographical and socioeconomic variations in observed travel durations of 0.5–2 h. Affluent families benefit from greater resources, access to expedited transportation, and gaining advantageous healthcare access. Conversely, economically disadvantaged families may experience prolonged travel times, exacerbating healthcare disparities [33, 34].

Residential location significantly impacts maternal health services, with rural women having 50% fewer ANC visits and higher rates of non‐institutional deliveries, indicating urban women’s superior access to ANC and institutional delivery services. Regardless of travel distance, there is a common threshold of attending at least three ANC visits, showcasing a baseline engagement in ANC across diverse geographies. Notably, in urban areas, women facing over two hours of travel time often exceed this baseline, possibly reflecting a commitment to ANC despite distance challenges, suggesting additional supportive factors, such as heightened awareness or resource access. While prior research identifies urban residency as a predictor of improved maternal health service use [27], the Afghan context indicates a shifting dynamic, with increasing healthcare demand in urban areas surpassing available supply.

The healthcare system’s structure, reliant on donor‐dependent BPHS for primary care and EPHS for higher‐level care, leads to notable service access disparities [35]. Fluctuating funding exacerbates rural service limitations and urban facility strains, causing overcrowding and reduced quality care [35, 36]. These systemic issues, coupled with infrastructure and economic challenges, highlight the fragile state of healthcare, which is susceptible to political shifts, exemplified by the collapse of the internationally assisted Afghan government [37].

Climate disasters and geopolitical crises and disease outbreaks profoundly affect healthcare access, exacerbating travel time challenges [30, 38]. For example, the October 2023 earthquake in Herat province destroyed 40 health facilities, hindering access for approximately 7500 women in the affected areas in need of services [39]. These events underscore the necessity of not only the availability but also the acceptability and quality of maternal healthcare services, particularly for minority and marginalized groups who are disproportionately affected by such crises. The Countdown report underscores the importance of equity, noting that the most vulnerable and marginalized individuals in Afghanistan have the lowest utilization rates [18]. The Lancet’s recent publication on maternal health underscores the urgency of addressing these disparities with tailored actions [40].

Health systems in fragile and conflict‐affected (FCA) settings face challenges in prioritizing actions, allocating resources, and managing sudden crises [41]. The 2018 survey occurred during a time of significant national and international support in Afghanistan, which contrasts the current situation with the impact of the COVID‐19 pandemic, a regime change, significant internal and external migration, and escalating poverty levels [42]. These factors have contributed to a deterioration in conditions since the survey, emphasizing an increased urgency for evidence‐based, targeted, and tailored interventions.

Cultural norms in Afghanistan require female healthcare providers, such as midwives, to deliver maternity services. However, recent restrictions on women’s educational bans significantly impede access to care and hinder the future supply of female healthcare professionals [29]. Additionally, travel restrictions for women without a male chaperone further compound these barriers [29]. Additionally, educational attainment, both for women and their families, is crucial for improving maternal health outcomes. Women’s educational attainment directly impacts their use of ANC and preference for institutional delivery. Understanding pregnancy risks through effective counseling and education empowers women to make informed choices and fosters proactive healthcare behavior [31]. However, educational barriers and limited health literacy leave many women uncertain about the quality and respect they deserve, emphasizing the need for improved health education programs [31, 37].

This empowerment extends beyond individual women: Husbands’ involvement in decision‐making correlates with increased institutional deliveries, and broader community and family engagement in maternal health education further boosts ANC utilization and facility births [17]. Integrating health education into these networks, especially in resource‐limited areas, builds a critical support system for better maternal care [42, 43]. This is further echoed by the study’s finding that women with access to information through the internet, TV, or radio are more likely to choose institutional deliveries. While such access may reflect higher socioeconomic status, it underscores the importance of making widespread and accessible health information a key driver of empowered choices for maternal health [31].

Alternative funding strategies, such as public‐private partnerships and community investment models, could provide the necessary support for these health services. Incentive‐based models, including healthcare vouchers accompanied by quality assurance measures, are worth exploring to encourage engagement with maternal health services. Additionally, the scaling up of technological solutions, like the promising mobile messaging for prenatal care, could significantly extend the reach and enhance the impact of health education. These methods could be complemented by the active participation of local organizations and professional associations, leveraging their on‐the‐ground insights and networks to ensure interventions are culturally aligned and locally supported.

### 4.1. Limitations and Strengths

There were some strengths in our study. First, we used data from a nationally representative survey, which may enhance the credibility of our results and enable the results to be generalized at the country level. Second, we used a survey questionnaire and study design that closely mirrored those used in demographic health surveys conducted in low‐ and middle‐income countries (LMICs). This methodology allows for the possibility that our results might be compared to those published in other LMICs. One limitation was the recall bias, as women recalled events from several months ago; women may have underreported the events and related attributes.

In addition, a limitation of our study is the exclusion of 4992 women with missing values on travel time, and this can raise a concern about selection bias in our findings. We examined and compared the distribution of characteristics of the 4992 women with the 1051 women that we included in the study, and we found that the distribution of data were somewhat similar between the two groups of women. This means that even if we did not use data from all eligible women, the chance of selection bias may not be considerable in our findings.

Another limitation of this study is the potential impact of Afghanistan’s evolving political situation since 2018 on its findings. Changes in health infrastructure, the economy, and particularly women’s access to health services could significantly alter the study’s applicability to the current context. As such, the present analysis should be interpreted with caution, acknowledging that the findings may not fully reflect the current realities faced by Afghan women.

## 5. Conclusion

The findings of this study underscore the significant association of travel time and residential location with the use of ANC and institutional delivery services by Afghan women. The importance of accessibility is evident, particularly in humanitarian settings, and is further complicated by the pronounced divide between urban and rural healthcare services. Our study calls for policies that go beyond reducing travel time to bridge gaps in maternal healthcare access and between socioeconomic groups.

## Author Contributions

Massoma Jafari, Muhammad Haroon Stanikzai, Essa Tawfiq, Sheena Currie, and Sabera Turkmani conceptualized the study. Essa Tawfiq and Muhammad Haroon Stanikzai analyzed the data. Massoma Jafari, Muhammad Haroon Stanikzai, Essa Tawfiq, and Sheena Currie drafted the manuscript. Massoma Jafari, Essa Tawfiq, Sheena Currie, Muhammad Haroon Stanikzai, Sabera Turkmani, Fatima Arifi, Faiza Rab, Hawa Kazemi, and Abdul Wahed Wasiq provided critical review and feedback to the revised versions of the manuscript.

## Funding

No funding was received for this research.

## Disclosure

All authors have reviewed and approved the final version of the manuscript submitted for publication.

## Conflicts of Interest

The authors declare no conflicts of interest.

## Supporting Information

S1: Baseline characteristics of women and other characteristics related to accessing health facilities.

## Supporting information


**Supporting Information** Additional supporting information can be found online in the Supporting Information section.

## Data Availability

The data used to support the findings of this study can be requested from the Afghanistan Ministry of Public Health (Email: info.access@moph.gov.af).
