# Supplementary material for: Association of Travel Time and Residential Location With the Use of Antenatal Care and Institutional Delivery Services in Afghanistan
Source: Obstet Gynecol Int. 2026 Jan 28;2026:1366466. doi: 10.1155/ogi/1366466 (PMC12851514; doi:10.1155/ogi/1366466)
Supplement: Supplementary file 1 — Supporting Information Additional supporting information can be found online in the Supporting Information section. [file OGI-2026-1366466-s001.docx]

| Supplementary table. Baseline characteristics of women and other characteristics related to accessing health facilities | | | | | | | | |
| --- | --- | --- | --- | --- | --- | --- | --- | --- |
|  |  | Reported travel time was not missing | | |  | Reported travel time was missing | | |
|  |  | deliveries | |  |  | deliveries | |  |
|  |  | Non-institutional | Institutional | Total |  | Non-institutional | Institutional | Total |
|  |  | n=298 | n=753 | n=1051 |  | n=1545 | n=3447 | n=4992 |
| Residential areas | |  |  |  |  |  |  |  |
|  | Urban | 43 (14.4%) | 196 (26.0%) | 239 (22.7%) |  | 201 (13.0%) | 1128 (32.7%) | 1329 (26.6%) |
|  | Rural | 255 (85.6%) | 557 (74.0%) | 812 (77.3%) |  | 1344 (87.0%) | 2319 (67.3%) | 3663 (73.4%) |
| Health worker who provided ANC services | | |  |  |  |  |  |  |
|  | Community Health Worker | 2 (0.7%) | 1 (0.1%) | 3 (0.3%) |  | 10 (0.7%) | 13 (0.4%) | 23 (0.5%) |
|  | Nurse | 0.0% | 4 (0.5%) | 4 (0.4%) |  | 9 (0.5%) | 37 (1.0%) | 46 (0.9%) |
|  | Doctor | 101 (33.9%) | 292 (38.8%) | 393 (37.4%) |  | 489 (31.7%) | 1046 (30.4%) | 1535 (30.7%) |
|  | Midwife | 195 (65.4%) | 456 (60.6%) | 651 (61.9%) |  | 1037 (67.1%) | 2351 (68.2%) | 3388 (67.9%) |
| Age of women | |  |  |  |  |  |  |  |
|  | 15-29 years | 173 (58.1%) | 416 (55.3%) | 589 (56.1%) |  | 873(56.5%) | 2119 (61.5%) | 2992 (59.9%) |
|  | 30-34 years | 72 (24.2%) | 168 (22.2%) | 240 (22.8%) |  | 386 (25.0%) | 761 (22.1%) | 1147 (23.0%) |
|  | 35-49 years | 53 (17.8%) | 169 (22.5%) | 222 (21.1%) |  | 286 (18.5%) | 567 (16.4%) | 853 (17.1%) |
| Education level of women | |  |  |  |  |  |  |  |
|  | No formal education | 239 (80.2%) | 575 (76.4%) | 814 (77.5%) |  | 1343 (86.9%) | 2576 (74.7%) | 3919 (78.5%) |
|  | Primary education | 47 (15.8%) | 112 (14.8%) | 159 (15.1%) |  | 149 (9.6%) | 494 (14.3%) | 643 (12.9%) |
|  | Secondary education | 12 (4.0%) | 66 (8.8%) | 78 (7.4%) |  | 53 (3.4%) | 377 (10.9%) | 430 (8.6%) |
| Woman knows danger sign | |  |  |  |  |  |  |  |
|  | No | 107 (35.9%) | 243 (32.3%) | 350 (33.3%) |  | 621 (40.2%) | 1117 (32.4%) | 1738 (34.8%) |
|  | Yes | 191 (64.1%) | 510 (67.7%) | 701 (66.7%) |  | 924 (59.8%) | 2330 (67.6%) | 3254 (65.2%) |
| Decision made for woman where to give birth | | |  |  |  |  |  |  |
|  | Herself | 177 (59.4%) | 193 (25.7%) | 370 (35.2%) |  | 866 (56.1%) | 877 (25.4%) | 1743 (34.9%) |
|  | Husband | 72 (24.2%) | 367 (48.7%) | 439 (41.8%) |  | 459 (29.7%) | 1752 (50.8%) | 2211 (44.3%) |
|  | In-laws | 24 (8.0%) | 165 (21.9%) | 189 (18.0%) |  | 167 (10.8%) | 734 (21.3%) | 901 (18.1%) |
|  | Others | 25 (8.4%) | 28 (3.7%) | 53 (5.0%) |  | 53 (3.4%) | 84 (2.4%) | 137 (2.7%) |
| Woman had miscarriage in the past | | |  |  |  |  |  |  |
|  | No | 192 (64.4%) | 501 (66.5%) | 693 (65.9%) |  | 1135 (73.5%) | 2543 (73.8%) | 3678 (73.7%) |
|  | Yes | 106 (35.6%) | 252 (33.5%) | 358 (34.1%) |  | 410 (26.5%) | 904 (26.2%) | 1314 (26.3%) |
| Access to media (radio, TV, internet) | | |  |  |  |  |  |  |
|  | No | 158 (53.0%) | 316 (42.0%) | 474 (45.1%) |  | 924 (59.8%) | 1421 (41.2%) | 2345 (47.0%) |
|  | Yes (almost every day) | 772 (5.8%) | 270 (35.9%) | 347 (33.0%) |  | 348 (22.5%) | 1243 (36.1%) | 1591 (31.9%) |
|  | Yes (once a week) | 63 (21.1%) | 167 (22.1%) | 230 (21.9%) |  | 273 (17.7%) | 783 (22.7%) | 1056 (21.2%) |
| Data from women with missing values on travel time were excluded from the main analysis, because reported travel time was the main predictor of the outcomes examined in this study. | | | | | | | | |
